# Comparison between two methods of working length determination and its effect on radiographic extent of root canal filling: a clinical study [ISRCTN71486641]

**DOI:** 10.1186/1472-6831-6-4

**Published:** 2006-02-11

**Authors:** L Smadi

**Affiliations:** 1 Assistant Professor, Endodontic department, Faculty of Dentistry, University of Jordan, Amman, Jordan

## Abstract

**Background:**

Obtaining a correct working length is critical to the success of endodontic therapy. Different methods have been used to identify this crucial measurement. The Aim of this clinical study was to compare the effect of working length determination using apex locator alone or in combination with working length radiograph on the apical extent of root canal filling.

**Methods:**

A total number of 66 patients, 151 canals were randomized into two groups, In group (I) working length was determined by apex locator alone, while in group (II) working length was determined by apex locator confirmed by working length radiograph, length of obturation was assessed, and the total number of radiographs was recorded. The data were analyzed using SAS system and T. tests were carried out. Statistical significance was considered to be P ≤ 0.05.

**Results:**

Sixty seven canals in group I were treated with a mean distance from the tip of root canal filling to radiographic apex -0.5 mm ± 0.5 and a mean of a total number of radiographs of 2.0, while in group II eighty four canals were treated with a mean distance from the tip of root canal filling to radiographic apex -0.4 mm ± 0.5 and a mean of a total number of radiographs of 3.2. There was no statistically significant difference in the mean distance from the tip of root filling to radiographic apex between group I and group II (P > 0.05).

**Conclusion:**

The practice of using electronic apex locator in the determination of working length is useful and reliable with no statistical difference of the radiographic extent of root canal filling when using apex locator alone or in combination with working length radiograph. Under the clinical conditions of this study, it is suggested that the correct use of an apex locator alone could prevent the need for further diagnostic radiographs for determination of working length. This method can be useful in patients who need not to be exposed to repeated radiation because of mental, medical or oral conditions.

## Background

One of the main concerns in root canal treatment is to determine how far working instruments should be advanced within the root canal, and at what point the preparation and obturation should be located [1,2].

The significance of the apical constriction in the root canal treatment is well recognized. It is generally accepted that the preparation and obturation should be at or short of the apical constriction [3].

Although radiography is the most commonly used diagnostic aid in endodontics, It can, at best, give an estimate of this histological structure and although clinically desirable, averages used to define the apical constriction from radiographic apex could lead to over or under filling [4].

In theory apex locator that locates the apical constriction may more accurately determines the physiological end-point of root canal therapy. The main shortcoming of early apex locators (erroneous reading with electrolytes) [5] was overcome by Kobayashi 1991 [6] with the introduction of ratio method. In this method two electric currents with different sine wave frequencies will have measurable impedances that can be measured and compared as a ratio regardless of the type of electrolyte in the canal. The capacitance of the root canal increases significantly at the apical constriction, and the quotient of the impedances reduces rapidly as the apical constriction is reached [7,8]. The Tri Auto ZX (J. Morita Co., Kyoto, Japan) is a cordless hand piece with an integrated apex locator and designed for rotary canal preparation with nickel titanium instruments based upon the same principle [9]. The Tri auto ZX has a reported accuracy similar to Root ZX of 95% [10] with the addition of some safety features such as auto-reverse when working length is reached [11].

It is believed that apex locators should not be considered as a replacement of radiographs [12] but can be considered as a good supplement to working radiographs that may improve length determination [13,14]and could potentially reduce the number of diagnostic radiographs required for working length determination [15].

Although, accurate measurements were achieved using modern apex locators [10,16-21], today, the common practice is to verify electronic measurement by radiograph and to refer to the radiograph as the most reliable source for root length determination [22,23].

The purpose of this clinical study was to compare the effect of working length determination using apex locator alone or in combination with working length radiograph on the apical extent of root canal filling.

## Methods

A total number of 66 patients with 151 canals were treated at endodontic department, Jordan University Hospital. Informed written consent was obtained from each patient following ethical committee approval from the deanship of academic research at the University of Jordan.

The patients were 30 males and 36 females between 12–65 years old with a mean of (35.3 ± 15.5). Patients that used heart pacemakers were excluded. All patients were referred for endodontic treatment and were treated by the author. A data sheets were used for recording the patient's profile, chief complaint, history of present illness, medical history, dental history, investigations, diagnosis and treatment planning, vitality of the tooth on access as determined by bleeding, working length measurements, procedures done, total number of radiographs, number of radiographic retakes, total number of visits, the incidence of flare-ups (that require unscheduled endodontic intervention), with all of the corresponding radiographs (film mounts, Dentsply RINN, UK).

A preoperative periapical radiograph (AGFA, Dentus M2, Heraeus, Kulzer, Germany) placed in a film holder (Hawe Super-Bite, Kerr Hawe SA, Switzerland) was taken (Trophy Vincennes France, HT KV 65, mA 8). After administration of local anesthesia (Persocaine-E^® ^Daron Pakhsh, Iran) pulp cavity was accessed using a straight fissure diamond bur in a high speed contrangle hand piece under abundant water spray followed by small round bur in a slow speed hand piece, after initial access the tooth was isolated using rubber dam. The walls of the access cavity were adjusted using endo Z bur (Dentsply Maillefer, Ballaigues, Switserland), the entrances of the root canals were irrigated with 2.5 % sodium hypochlorite solution and dried by aspiration, no attempt was made to clean debris or pulp tissue remnants prior to introducing a size 10 k-file (Dentsply Maillefer, Ballaigues, Switserland) into the canals. The Tri Auto ZX^® ^was used on EMR Mode- Electronic measurement of a root canal based on manufacturer's recommendations (group I). The lip-clip (contrary electrode) was placed in the corner of the patient's mouth and the file holder was attached onto the shaft of the hand file. The size 10 K-file was advanced apically into the canal until the beeping sound and the light emitting diode (LED) marked APEX on the panel began to glow, indicating that the tip of the file had reached the anatomical end of the canal in the periodontium. The file was withdrawn with a slow counterclockwise turn until the red LED APEX light went out, suggesting that the tip of the file was at the predetermined length of the apical constriction. At that time, the 0.5 mm green LED light illuminated and a rapid tone was heard. The distance from the file tip to the silicone stop was then measured to an accuracy of 0.5 mm (Minifix, VDW, Germany) and registered as working length. In group (II) with a size 10 or 15 K-files set on a pre-estimated electronic length inside root canals a working length radiograph (AGFA, Dentus M2, Heraeus, Kulzer, Germany) placed in a film holder (Hawe Endo-bite (anterior/posterior), Kerr Hawe SA, Switzerland) was taken (Trophy Vincennes France, HT KV 65, mA 8). The radiographic length was then recorded. If any discrepancy between the electronic length and the radiographic length was found then a decision was made on the appropriate adjustment based on both the radiographic picture aided by the electronic measurements. Radiographs were repeated if root apices were not clear.

The patients were randomized into group I and group II alternately as they referred for treatment.

Cleaning and shaping was done using flexofiles size 15–40 (Dentsply Maillefer, Ballaigues, Switserland) in a step back technique, followed by nickel titanium protaper rotary files (Dentsply Maillefer, Ballaigues, Switserland) in Tri Auto ZX hand piece pre set on the manual mode, accompanied by copious irrigation of sodium hypochlorite 2.5%, before obturation the canal was thoroughly dried, AH26 was the sealer used with hot lateral condensation of conventional gutta percha (size medium, medium-fine, Meta Dental Co Ltd, Korea) using hand spreader (A30 Maillefer, Switserland) heated in a bead sterilizer. A postoperative periapical radiograph (AGFA, Dentus M2, Heraeus, Kulzer, Germany) placed in a film holder (Hawe Super-Bite, Kerr Hawe SA, Switzerland) was taken (Trophy Vincennes France, HT KV 65, mA8). Root canal treatment was finished in one to three visits according to pathological status, time available, the difficulty of the case and patient cooperation.

Periapical post-obturation radiographs were examined on x-ray illuminator and by the aid of magnifying loupes 3.5 × (XL advantage, 42 cm working distance, 8.1 field diameter, Keeler, UK). Distances from the end of root canal filling to the radiographic apex were measured in millimeter to an accuracy of 0.5 mm twice at two separate occasions and the means were calculated and recorded.

The data were analyzed using SAS system and T. tests were carried out. Statistical significance was considered to be P ≤ 0.05.

## Results

A total of 67 canals in group I, in which an apex locator was used without a radiograph, showed a mean distance from the tip of root canal filling to radiographic apex of -0.5 mm and a mean of a total number of radiographs of only 2.0 was needed. While in group II, in which an apex locator was used in conjunction to a radiograph, a total of 84 canals showed a mean distance from the tip of root canal filling to radiographic apex -0.4 mm and a mean of a total number of radiographs of only 3.2, including a preoperative and a postoperative radiographs, was needed (table 1). There was no statistical significant difference in the mean distance from the tip of root filling to radiographic apex between group 1 and group II with P = 0.3224 (P > 0.05).

**Table 1:** The mean distance from the tip of root canal filling to radiographic apex and the mean total number of radiographs in group I and group II.

**Variable**	**Group I**			**Group II**		
	
	Number	Mean	SD	Number	Mean	SD
**Mean distance from tip of root canal filling to radiographic apex (mm)**	67	-0.5	0.5	84	-0.4	0.5
**Number of Radiographs (mean)**	67	2.0	0.1	84	3.2	0.5

SD = standard deviation

When root canals were divided into subgroups according to tooth position and pulpal status, similar results were obtained showing no statistical difference in the mean distance from the end of the root filling to radiographic apex between the two groups in all subgroups treated (table 2).

**Table 2:** Comparison of the mean distance from the tip of root canal filling to the radiographic apex in group I and group II in different subgroups.

**Variable**		**Group I**			**Group II**			**P value**
			
		**Number**	**Mean**	**SD**	**Number**	**Mean**	**SD**	
**Tooth Position**	**Premolar teeth**	17	-0.5	0.6	19	-0.3	0.5	0.341
	**Molar teeth**	50	-0.4	0.5	57	-0.3	0.5	0.381
	**Upper teeth**	36	-0.5	0.6	55	-0.3	0.5	0.270
	**Lower teeth**	32	-0.4	0.4	29	-0.4	0.3	0.963
**Tooth Status**	**Vital**	19	-0.6	0.6	24	-0.3	0.6	0.829
	**Non- vital**	48	-0.4	0.4	50	-0.4	0.4	0.152

The total number of radiographs which was needed during treatment was counted in the different subgroups and a mean of two radiographs and approximately three radiographs (3.1–3.4) was scored in group I and group II, respectively (table 3).

**Table 3:** The mean total number of radiographs in group I and group II in different subgroups.

**Variable**		**Group I**			**Group II**		
		
		**Number**	**Mean**	**SD**	**Number**	**Mean**	**SD**
**Tooth Position**	**Premolar teeth**	17	2.0	0	19	3.1	0.3
	**Molar teeth**	50	2.0	0.1	57	3.2	0.5
	**Upper teeth**	36	2.0	0	55	3.2	0.5
	**Lower teeth**	32	2.0	0.1	29	3.1	0.4
**Tooth Status**	**Vital**	19	2.0	0.2	24	3.4	0.7
	**Non- vital**	48	2.0	0	50	3.1	0.3

In order to test the effect of a preoperative status on the behavior of TAZX apex locator, the radiographic extent of root canal filling was compared in vital and non-vital canals (table 4). There was no statistical difference in the mean distance from the tip of root filling to radiographic apex between vital and non-vital canals P = 0.467 (P > 0.05).

**Table 4:** The mean distance from the tip of root canal filling to the radiographic apex in vital and non-vital canals.

**Variable**	**number**	**mean**	**SD**
**vital**	43	-0.5 mm	0.6
**Non-vital**	98	-0.4 mm	0.4

## Discussion

The use of the electronic methods for tooth length determination has progressed substantially and has gained popularity in recent years [10,24].

Several studies were previously executed investigating accuracy of a given apex locator. In vivo studies in which teeth to be extracted were used to compare electronic length to different target points such as apical foramen, apical constriction or radiographic apex [10,17-19,25].

In vitro studies used electro conductive materials like alginate, gelatin, agar or saline to simulate the clinical situations [16,20,21,26,27].

Unlike those studies, the present study is a clinical study that incorporates all errors which may occur in the mouth [23,28].

This study showed that similar results of radiographic extent of root canal fillings with no statistical difference between the two methods using apex locator alone or in combination with working length radiograph. These findings suggest that apex locator can potentially replace, in many instances, the classic radiographic method for tooth length determination and that the correct use of a calibrated apex locator would prevent the need for further diagnostic radiographs for working length determination. This suggestion would comply with As Low As Reasonably Achievable principle (ALARA) [29,30] particularly when taking into account the limitations of radiographs [5,31,32] in one hand and the reported great accuracy of modern apex locators and reliability over radiographs on the other hand [10,16-21]. This suggestion disagree with Hoer and Attin (2004) [23] who suggested that working length determination should be carried out using a combination of apex locator and radiography, although their study showed that apex locators were able to identify the interval "apical constriction to major foramen " with a high degree of success. Nevertheless, the latter approach has other advantages over using apex locator alone including better understanding of root canal morphology, root canal curvature and portal of apical terminus.

The performance of the apex locator showed similar reliability obtained in previous

Studies [10,17-21] in both tested methods in correlation to apical extent of root canal filling to the radiographic apex within a tolerance level of ± 0.5 mm from radiographic apex.

According to Fouad and Reid 2000 [13] a clinically acceptable length of obturation has been achieved in both tested methods (0–2 mm short of the apex).

In agreement with previous studies [10,25] the preoperative status of the pulp did not appear to affect the behavior of the TAZX apex locator in correlation to the apical extent of root canal filling using both methods of working length determination which disagrees with other previous observation which indicate that the Apex Finder showed higher accuracy in vital canal than in necrotic canals [34].

The results of the present study also showed that electronic predetermination of working length prior to working length radiograph resulted in a total number of a mean of approximately three radiographs including pre-operative and post-operative radiographs which means that approximately a zero retake was needed. This agrees with previous study which concluded that adjunctive use of an apex locator reduces the number of diagnostic radiographs required for working length determination [15]. Another study showed that adjunctive use of an apex locator did reduce the number of working radiographs in anterior and premolar teeth, but not in molars [13]. A further reduction of radiation dose during endodontic therapy has been suggested by combining an apex locator and a digital imaging system [35]. This currently introduced digital radiography has not been shown to exceed conventional radiography in quality, but is useful for its speed of image acquisition, reduced patient irradiation and the possibility of enhancing or editing image [36].

It must be emphasized that the use of apex locator alone without a preoperative and postoperative radiograph is not a recommended practice due to the large variation in tooth morphology and medico-legal record keeping requirements [5]. A recent case report highlighted the requirement for a preoperative radiograph where an apex locator was initially used. The patient was pregnant and subsequent failure to detect an anomalous root lead to the failure of treatment [28]. The results of this study needs to be verified in a more representative number of canals with a varying degree of clinical conditions.

## Conclusion

The practice of using electronic apex locator in the determination of working length is useful and reliable with no statistical difference of the radiographic extent of root canal filling when using apex locator alone or in combination with working length radiograph. Under the clinical conditions of this study, it is suggested that the correct use of an apex locator alone could prevent the need for further diagnostic radiographs for determination of working length. This method can be useful in patients who need not to be exposed to repeated radiation because of mental, medical or oral conditions.

## Competing interests

The author(s) declare that they have no competing interests.

## Pre-publication history

The pre-publication history for this paper can be accessed here:


